# ScAlN Film-Based Piezoelectric Micromechanical Ultrasonic Transducers with Dual-Ring Structure for Distance Sensing

**DOI:** 10.3390/mi14030516

**Published:** 2023-02-23

**Authors:** Yuchao Zhang, Bin Miao, Guanghua Wang, Hongyu Zhou, Shiqin Zhang, Yimin Hu, Junfeng Wu, Xuechao Yu, Jiadong Li

**Affiliations:** 1 Suzhou Institute of Nano-Tech and Nano-Bionics, Chinese Academy of Sciences, Suzhou 215123, China; 2 Key Laboratory of Multifunctional Nanomaterials and Smart Systems, Chinese Academy of Sciences, Suzhou 215123, China; 3 School of Electrical and Mechanical Engineering, Changchun University of Science and Technology, Changchun 130022, China; 4 School of Aeronautics, Nanjing University of Aeronautics and Astronautics, Nanjing 211106, China

**Keywords:** piezoelectric micromechanical ultrasonic transducer (pMUT), distance sensors, scandium-doped aluminum nitride (ScAlN), dual-ring structure

## Abstract

Piezoelectric micromechanical ultrasonic transducers (pMUTs) are new types of distance sensors with great potential for applications in automotive, unmanned aerial vehicle, robotics, and smart homes. However, previously reported pMUTs are limited by a short sensing distance due to lower output sound pressure. In this work, a pMUT with a special dual-ring structure based on scandium-doped aluminum nitride (ScAlN) is proposed. The combination of a dual-ring structure with pinned boundary conditions and a high piezoelectric performance ScAlN film allows the pMUT to achieve a large dynamic displacement of 2.87 μm/V and a high electromechanical coupling coefficient ($k_{t}^{2}$) of 8.92%. The results of ranging experiments show that a single pMUT achieves a distance sensing of 6 m at a resonant frequency of 91 kHz, the farthest distance sensing registered to date. This pMUT provides surprisingly fertile ground for various distance sensing applications.

## 1. Introduction

Ultrasonic transducers can provide accurate distance information independent of object color and ambient light intensity, and they play an important role in distance sensing applications, such as parking assistance [1], robot obstacle avoidance [2], and presence detection [3]. However, conventional bulk piezoelectric transducers are large, power-hungry, and incompatible with integrated circuits, which makes them impractical for miniaturized and integrated mobile applications. Piezoelectric micromechanical ultrasonic transducers (pMUTs) based on microelectromechanical systems (MEMS) technology have certain advantages such as small size, low power consumption, high sensitivity, and high integration [4,5,6,7], thus being effective solutions for expanding the application of piezoelectric ultrasonic transducers.

The two main piezoelectric materials used in PMUTs are lead zirconium titanate (PZT) and aluminum nitride (AlN). Among them, PZT has a high piezoelectric coefficient (*e*_31*,f*_ ≈ −13.1); thus, it can provide a large emitted sound pressure. However, the lower dielectric constant of PZT (*ε*_33_ ≈ 854) makes its reception sensitivity lower [8]. Although the piezoelectric coefficient of AlN is much lower than that of PZT, its ratio of piezoelectric coefficient to dielectric constant and quality factor are better than that of PZT, which is very suitable for piezoelectric sensors [9]. In addition, AlN has the advantage of low deposition temperature (≤300 °C) and compatibility with complementary metal oxide semiconductor (CMOS) fabrication processes compared to PZT [10,11,12,13]. Therefore, AlN pMUTs are attractive for ranging.

Over the past two decades, AlN pMUT-based ultrasonic rangefinders have made increasing breakthroughs in improving the distance sensing. For instance, in 2011, Przybyla et al. proposed a pMUT with an elastic layer of 1 µm thick silica to achieve distance sensing up to 1.3 m [14]. In 2019, Alasatri et al. fabricated a two-electrode pMUT. The two-electrode design isolates the transmit and receive within a single pMUT paths, avoiding the frequency mismatch problem common to pMUT array designs, and achieves a sensing range of 1.5 m [15]. In 2020, Chrip made a breakthrough. They used advanced ultrasonic DSP algorithms and acoustic interfaces to enable the pMUT to achieve a sensing range of up to 5 m [16]. However, for other potential applications using conventional air-coupled ultrasonic sensors, such as automotive parking assistance and robotic proximity sensors, existing pMUTs have yet to provide sufficient pressure output to have adequate sensing range. 

In recent years, there have been attempts to use Sc-doped AlN to enhance the performance of pMUTs. Scandium aluminum nitride (Sc_x_ Al_1−x_ N) retains many of the properties of AlN (e.g., CMOS compatibility, easy deposition/etching, and lead-free) [17,18] but has a higher piezoelectric coefficient and similar dielectric constant [19] compared to pure AlN, resulting in better transmission performance. Yuri et al. designed a 2 × 2 Sc_0.36_Al_0.64_N pMUT array, which achieves a high transmission pressure of 105 dB SPL at a distance of 10 cm and produces only 30 dB of attenuation at a range of 2 m [18]. Haolin et al. designed a 14 Sc_0.2_Al_0.8_N pMUT array and obtained its maximum range of 6.8–8.4 m (0% air humidity–100% air humidity) at a resonant frequency of 66 kHz through theoretical calculations [20]. However, the maximum sensing range of the single ScAlN pMUT is yet to be further evaluated.

In this paper, a pMUT based on a dual-ring structure of Sc_0.25_Al_0.75_N is proposed to increase the sensing distance. This pMUT achieves a large amplitude of 2.87 μm/V and a high electromechanical coupling coefficient of 8.92% ($k_{t}^{2}$). The ranging capability of this pMUT was evaluated with a single device that is both a transmitter and a receiver, achieving a distance sensing of 6 m at a resonant frequency of 91 kHz, i.e., the farthest sensing distance up to date. Consequently, our results indicate that this pMUT becomes the most promising alternative to conventional bulk piezoelectric transducers, thus playing an important role in various distance sensing applications.

## 2. Theory and Modeling

### 2.1. Transducer Description

**Figure 1:**
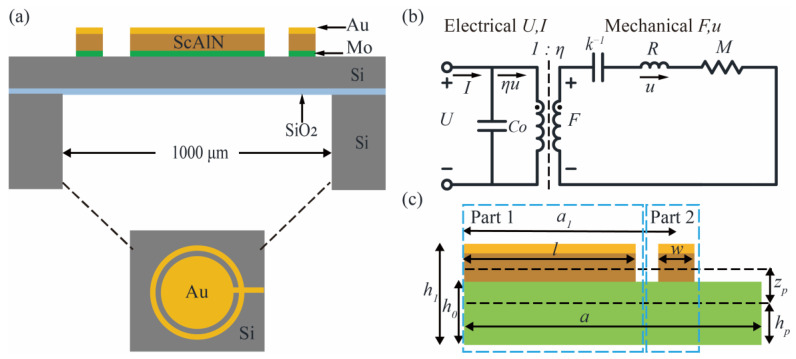
(**a**) Cross-section and top schematic of the pMUT; (**b**) equivalent circuit model of a typical sensor device; (**c**) schematic diagram of the boundary structure.

Figure 1a shows the structure of the novel pMUT reported in this paper. The sensing diaphragm consists of a Mo/ScAlN/Au sandwich prepared on SOI sheets with thicknesses of 0.2 μm/1 μm/0.2 μm, respectively. The sandwich is divided via etching into a central circular membrane structure and an outer ring structure. The width of the outer ring is 20 μm, whereas the width of the isolation trench is 70 μm. The internal structure covers 67% of the radius of the diaphragm sheet, where the diaphragm radius is defined by a back-side release etching process. The electric field generated by the voltage applied between the Au and Mo electrodes causes transverse stresses in the ScAlN layer, which leads to the bending of the film, thus resulting in pressure waves.

The ScAlN has higher piezoelectric properties than AlN and, thus, can significantly improve the performance of pMUTs. The piezoelectric coefficients *e*_31*,f*_ is greatly increased for 25% Sc-doped AlN, while the dielectric constants *ε*_33_ is only slightly increased [21,22,23]. The pMUT reception sensitivity (S_R_) is defined as the proportion *e*_31*,f*_*/ε*_33_, whereas the electromechanical coupling coefficient $k_{t}^{2}$ is proportional to $e_{31,f}^{2}/\varepsilon_{33}$; therefore, the S_R_ of the ScAlN-based pMUT is about 12 times higher than PZT pMUT, and the $k_{t}^{2}$ is about twice higher than PZT and AlN pMUTs [24].

### 2.2. Equivalent Circuit Model

The pMUT is an electromechanical device in which electrical and mechanical energy is exchanged through a bidirectional coupling between the stress and the electric field in the piezoelectric material. The operation of the sensor is simulated using an equivalent circuit model (Figure 1b; the electrical domain is shown on the left and the mechanical domain is shown on the right). In the mechanical domain, the force *F* is equivalent to the voltage, and the velocity *U* is equivalent to the current. The equivalent circuit parameters are mechanical mass *M*, mechanical resistance *R*, mechanical stiffness *k*, and electromechanical conversion factor *η*. The *η* relates the voltage applied to the membrane to the force exerted by the membrane, i.e., *η = F/U*. Under the sinusoidal drive, the equation of motion of the pMUT can be expressed as follows [25]:

(1) $$j\omega Mu+Ru+\frac{ku}{j\omega }=F.$$

The mechanical stiffness *k* and mechanical mass *M* of pMUT can be deduced from the conservation of energy. For the convenience of calculation, the model is divided into two parts (Figure 1c): inside (circular membrane part 1) and outside (outer ring part 2); the mechanical stiffness is **1*/k = *1*/k*_1_ *+ *1*/k*_2_, and the mechanical mass is *M = M*_1_ *+ M*_2_. In the boundary structure (Figure 1c), a is the radius of the diaphragm, *l* is the radius of the piezoelectric layer and the top electrode in part 1, *w* is the width of part 2, *a*_1_ is the average radius of part 2, *h*_0_ is the thickness of the structural layer, *h*_1_ is the thickness of the structural and piezoelectric layers, *h_p_* is the distance from the bottom layer to the neutral axis, and *z_p_* is the distance from the center of the top layer to the neutral axis. The position of the neutral axis *z_NA_* is

(2) $$z_{NA}=\frac{\sum_{n=1}^{N}t_{n}z_{n}Y_{11}^{\prime }}{\sum_{n=1}^{N}t_{n}Y_{11}^{\prime }},$$

where *t_n_* is the thickness of the n-layer film, *z_N_* is the position of the *n*-th layer midplane relative to the bottom, and $Y_{11}^{\prime }$ is Young’s modulus. The etched grooves between parts 1 and 2 form the simple support boundary. The vibrational shapes of the simply supported thin plate [26] and the piezoelectric thin ring [25], respectively, can be approximated as

(3) $$\varphi_{1}\left(r\right)=1-1.741{\bar{r}}^{2}+0.7296{\bar{r}}^{4},$$

(4) $$\varphi_{2}\left(r\right)={\left[1-{\left(\frac{2\left(r-a_{1}\right)}{a}\right)}^{2}\right]}^{2},$$

where $\bar{r}=l/r$ is the normalized radial coordinate. Therefore, the mechanical stiffness *k* and mechanical mass *M* are

(5) $$k_{1}=\frac{D}{a^{2}}I_{elastic},$$

(6) $$M_{1}=2\pi \rho_{m}\int_{0}^{1}\varphi^{2}\left(\bar{r}\right)\bar{r}d\bar{r},$$

(7) $$k_{2}=\frac{2\pi w\left(h_{1}-h_{0}\right)}{s_{11}^{E}a_{1}},$$

(8) $$M_{2}=2\pi \rho_{m}a_{1}w\left(h_{1}-h_{0}\right),$$

(9) $$D=\frac{1}{3}\sum_{1}^{N}Y_{11}^{\prime }\left(\bar{h_{n}^{3}}-\bar{h_{n-1}^{3}}\right)$$

(10) $$I_{elastic}=2\pi \int_{0}^{1}\left[{\left(\frac{\partial^{2}\varphi }{\partial {\bar{r}}^{2}}{)}^{2}+\frac{2v}{\bar{r}}\frac{\partial^{2}\varphi }{\partial {\bar{r}}^{2}}\frac{\partial \varphi }{\partial \bar{r}}+{\left(\frac{1}{\bar{r}}\frac{\partial \varphi }{\partial \bar{r}}\right)}^{2}\right)}^{2}\right]\bar{r}d\bar{r},$$

where *ρ_m_* is the density, *I_elastic_* is the integral of strain, $s_{11}^{E}$ is the elastic flexibility matrix coefficient of the piezoelectric layer, *D* is the flexural stiffness, and $\bar{h_{n}}=h_{n}-z_{NA}$ is the distance from the top of the *n*-th level to the neutral axis. The electromechanical coupling coefficient is derived from the coupling energy *U_piezo_*. When *t* ≪ *l*,

(11) $$U_{piezo}=\frac{1}{2}M_{P}I_{piezo}sA_{0},$$

(12) $$I_{piezo}=2\pi \int_{0}^{1}\left(\frac{\partial^{2}\varphi }{\partial {\bar{r}}^{2}}+\frac{1}{\bar{r}}\frac{\partial \varphi }{\partial \bar{r}}\right)\bar{r}d\bar{r},$$

(13) $$\eta =\pi e_{31,f}z_{p}{\bar{r}}^{2}\left(2.9148\bar{r}-3.482\right),$$

where *Mp* is the piezoelectric bending moment, *Ielastic* is the strain integral in the electrode region, *A*_0_ is the displacement of the membrane, and *e*_31*,f*_ is the effective piezoelectric coefficient. The DC vibration amplitude is estimated from Equations (4), (6) and (9) as follows:

(14) $$w_{dc}=\frac{\eta U}{k}=\frac{\frac{1}{2}e_{31,f}z_{p}UI_{piezo}}{k_{1}+k_{2}}.$$

Compared with the pMUT of conventional structure [27], the electromechanical conversion factor improves about 1.2-fold, while the DC vibration amplitude improves almost twofold.

### 2.3. Fabrication Process Flow

**Figure 2:**
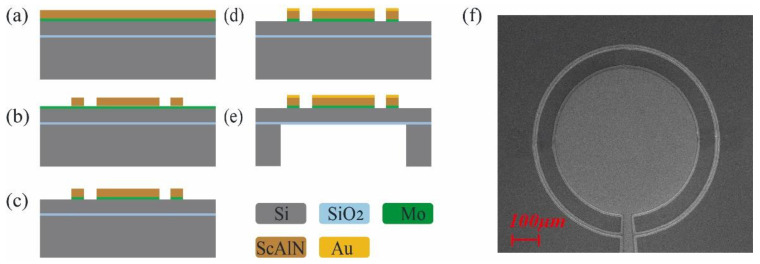
(**a**–**e**) The pMUT fabrication process flow; (**f**) SEM image of the top of the pMUT.

Figure 2a–e show the flowchart of the fabrication process for pMUT. The process starts with a ScAlN/Mo/SOI (25% Sc-doped AlN) wafer fabricated by Shanghai Normal University (Figure 2a), where the SOI wafer has a device silicon layer thickness of 5 μm and a buried oxygen layer thickness of 0.2 μm. Firstly, the ScAlN film is patterned and etched by ion beam etching (IBE) (Figure 2b). Since the Mo electrode layer is relatively thin, the time for etching the piezoelectric layer must be strictly controlled. Next, the electrode Mo under the piezoelectric film is patterned using plasma etching while retaining the external pins (Figure 2c). Subsequently, 200 nm thick gold is sputtered as the top electrode, with the diameter of the top electrode being slightly smaller than the diameter of the ScAlN film to prevent contact with the bottom electrode (Figure 2d). Lastly, the backside is etched by deep reactive ion etching (DRIE), and the septum is released to define the pMUT radius (Figure 2e). Here, 200 nm silica is used as an etch-stopping layer. The final fabricated pMUT cell with 500 µm radius is shown in Figure 2f. Compared to the manufacturing process of a conventional structured pMUT, no additional steps are added to the manufacturing of this pMUT, making the pMUT simple and suitable for mass processing and production.

## 3. Characterization and Discussion

### 3.1. Dynamic Characterization 

**Figure 3:**
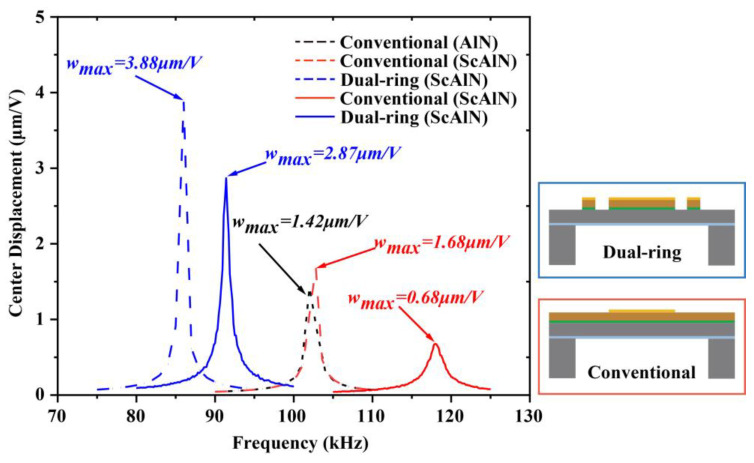
Simulated and tested frequency response curves of pMUT. The dashed line is the COMSOL simulation, and the solid line is the result of LDV test. The blue line shows the ScAlN pMUT with the dual-ring structure, the red line shows the ScAlN pMUT with the conventional structure, and the black line shows the AlN pMUT with the conventional structure.

The frequency response of the pMUT is shown in Figure 3. The dashed line is the pMUT frequency response curve simulated by COMSOL Multiphysics, whereas the solid line is the pMUT frequency response curve measured using a laser Doppler vibrometer (LDV, VIB-E-400, Polytec, Karlsruhe, Germany). The simulations and experiments use pMUTs with the same radius. The peak vibration amplitude at resonance is 1.42 μm/V for the pMUT with a conventional structure using AlN as the piezoelectric layer, and 1.68 μm/V for the ScAlN pMUT with a conventional structure at the resonance frequency. The difference in amplitude between ScAlN and AlN is due to the decrease in hardness and increase in piezoelectric coefficient brought about by Sc alloying. The change in stiffness and mass caused by the dual-ring structure increased the vibration amplitude by 131% to 3.88 μm/V. The LDV test results show that the amplitude of the dual-ring ScAlN pMUT is about four times higher than that of the conventional structured ScAlN pMUT.

The reason for the discrepancy between simulated and experimental results is the presence of residual stresses. The residual stress in the film strongly affects the frequency and displacement of the pMUT [28]. Additionally, the nonuniform etching of the back cavity is another reason to consider. During the release of the overhanging film, the silicon oxide, which is the etch stopping layer, is transition etched, and this leads to an increase in the surface roughness of the silicon oxide layer, which causes a change in the center point displacement and frequency [14].

### 3.2. Electrical Characterization 

**Figure 4:**
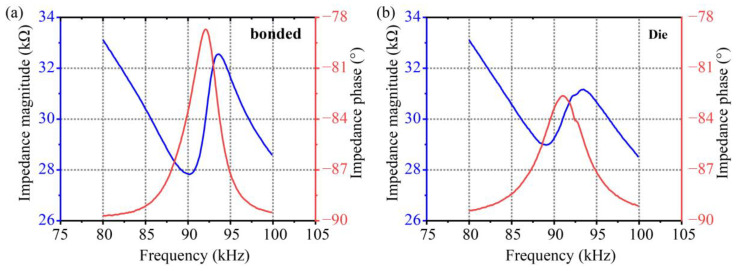
Measured electrical impedance values. (**a**) Bonded pMUT with resonant frequency *f*_0_ = 92 kHz, $k_{t}^{2}$ = 8.92%; (**b**) PMUT die with resonant frequency *f_0_* = 91 kHz, $k_{t}^{2}$ = 10.16%.

The impedance measurements of the bicyclic ScAlN pMUT were performed in air using a precision impedance analyzer (MICRO TEST 6632), as shown in Figure 4a,b. The electromechanical coupling coefficient was calculated as follows [29]:

(15) $$k_{t}^{2}=\frac{\pi^{2}}{4}\frac{f_{r}}{f_{a}}\frac{f_{a}-f_{r}}{f_{a}}.$$

Figure 4a shows the impedance value of a pMUT bonded to a printed circuit board (PCB) with epoxy resin, with values of 90.15 kHz and 93.67 kHz for the resonant frequency *f_r_* and the anti-resonant frequency *f_a_*, respectively, resulting in $k_{t}^{2}$ of 8.92%, which is about 370% larger compared to the bimorph, pinned pMUT and 743% larger compared to the unimorph, clamped pMUT in [28]. The electromechanical coupling coefficient is increased by about 350% compared to that of Sc_0.2_Al_0.8_N pMUT in reference [20]. The scandium doping and the dual-ring structure lead to a significant increase in the $k_{t}^{2}$, which increases the energy conversion efficiency, resulting in a larger output sound pressure.

Figure 4b shows the impedance values of the pMUT chip measured using the probe connected to the impedance analyzer with values of 89.45 kHz and 93.47 kHz for the resonant frequency fr and anti-resonant frequency fa, respectively, resulting in an electromechanical coupling factor of 10.16%. The electromechanical coupling factor of the pMUT die is 14% higher compared to the bound pMUT. This is due to the additional capacitance loss caused by the parasitic capacitance introduced when the pMUT is encapsulated on the PCB, as well as the power loss caused by the parasitic capacitance of the ranging circuit [30]. Reducing parasitic capacitance can effectively improve electromechanical conversion efficiency, thus enhance the device performance. 

### 3.3. Performance of Rangefinding

**Figure 5:**
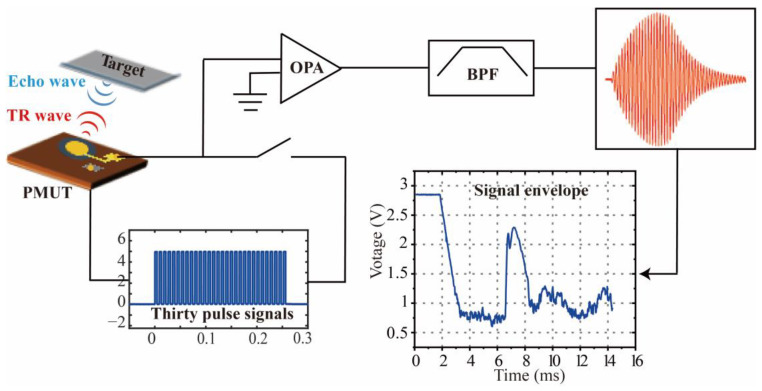
Block diagram of distance sensing system.

**Figure 6:**
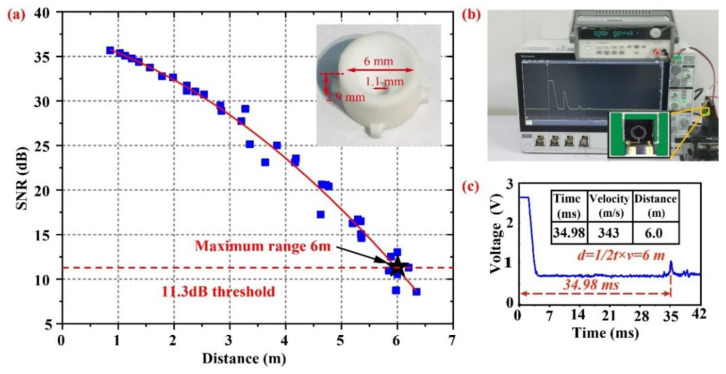
(**a**) SNR versus distance: at the threshold of 11.3 dB, the maximum range of this pMUT is 6 m(inset: the horn); (**b**) the distance measuring experimental device; (**c**) the envelope curve of the echo signal during distance measurement.

The block diagram of the distance sensing system is shown in Figure 5. The system consists of operational amplifiers, bandpass filters, and transceiver signal switching integrated on a single PCB. When the system is in transmit mode, the system sends out 30 bursts to excite the pMUT at its resonant frequency (91 kHz), thus resulting in a pressure wave. Subsequently, the system switches to receive mode, where the pMUT receives the sound waves and converts them into a voltage signal. An operational amplifier (OPA) at the front end amplifies the signal, followed by a bandpass filter (BPF) to filter the out-of-band noise from the amplifier. Finally, the returned signal is displayed on an oscilloscope. The oscilloscope displays the voltage amplitude of the echo signal and the system noise, as well as the time of flight of the ultrasonic waves. By extracting the time at which the amplitude of the maximum echo signal is located, the distance to the obstacle can be calculated. The experimental setup for distance measurement is shown in Figure 6b. The pMUT is bound to a PCB that is mounted on an adjustable moving platform. An external power supply powers the system at 5 Vpp, and the signal is output to an oscilloscope. The pMUT used in the experiments is shown in the inset in Figure 6b. The distance measurement target is a fixed and flat wall, the moving platform realizes the scanning of the pMUT in the horizontal direction, and the ultrasonic echo signal is displayed on the oscilloscope in the form of an envelope curve. The envelope curve of the echo signal is shown in Figure 6c. The time of flight of the ultrasonic wave is 34.98 ms; thus, the sensing distance is 6 m. 

A horn was used to enhance the radiated power during the ranging. The acoustic radiated power can be expressed as follows [25]:

(16) $$\bar{W}=\frac{1}{2}ZS^{2}u_{a}^{2},$$

where *u_a_* is the velocity amplitude of the sound source, *Z* is the impedance, and *S* is the area. The acoustic impedance of pMUT is calculated as follows [31]:

(17) $$Z_{pmut}=\frac{\rho_{0}c_{0}}{A_{m}}\left(1-\frac{2J_{1}\left(2ka\right)}{2ka}+i\frac{2K_{1}\left(2ka\right)}{2ka}\right),$$

where *J*_1_ and *K*_1_ are the first-order Bessel and Struve functions, *λ* is the wavelength, and *A_m_* is the effective vibrational area of the pMUT. The real part of *Z_pmut_* acts as a resistance, equivalent to mechanical damping, and the real part of *Z_pmut_* reaches a maximum value *Z*_0_ *= ρ*_0_*c*_0_*/A_m_*. The acoustic impedance of the throat of the horn is expressed as follows [25]:

(18) $$Z_{a0}=\frac{\rho_{0}c_{0}}{S_{0}}\left[\frac{Z_{al}\cos\left(\gamma l+\theta \right)+j\frac{\rho_{0}c_{0}}{S_{l}}sin\gamma l}{\frac{\rho_{0}}{S_{l}}\cos\left(\gamma l-\theta \right)+jZ_{al}sin\gamma l}\right],$$

where *S*_0_ is the area of the throat, and *Z_al_* is the acoustic impedance of the horn outlet. When *ka_l_* > 5, $Z_{al}\approx \frac{\rho_{0}c_{0}}{S_{l}}$, where *S_l_* is the area of the mouth. From Equations (16)–(18), the radiated power *W_al_* of the horn mouth is greater than the radiated power *W_pmut_* of the pMUT; thus, the use of the horn can effectively improve the acoustic output of the pMUT. Chrip [16] and Songsong Zhang et al. [20] designed and employed in previous pMUT ranging studies the acoustic interface, which enhances the acoustic output of the pMUT. Here, we use one with a length of 2.9 mm, a throat diameter of 1.1 mm, and a mouth diameter of 6 mm, as shown in Figure 6a.

The maximum range is determined by the minimum signal-to-noise ratio (SNR) required to reliably detect received pulses and reject false alarms due to noise. The SNR of the system can be expressed as

(19) $$\mathrm{SNR}=20log_{10}\left(\frac{A_{signal}}{A_{noise}}\right),$$

where *A_signal_* is the amplitude of the echo signal, and *A_noise_* is the amplitude of the noise. To prevent false alarms, the SNR threshold must be set high enough. The average time between false alarms is as follows [32]:

(20) $$t_{fa}=\frac{1}{BW}e^{\frac{V_{Th}^{2}}{\sigma^{2}}},$$

which results in $V_{Th}^{2}$/σ^2^ being 11.3 dB when *t_fa_* was determined to be 30 min. Following this definition, we plotted the distance versus signal-to-noise ratio for this pMUT, as shown in Figure 6a. When the signal-to-noise ratio threshold is set to 11.3 dB, the maximum sensing distance of this pMUT is 6 m at a drive voltage of 5 Vpp. The high performance at low voltages gives the possibility of integration by CMOS circuits to obtain long-range sensing without a special boost process, which helps reduce unnecessary energy loss. The low-voltage drive also avoids the rapid deterioration of emission performance that can be caused by high drive voltages, enhancing system reliability. Of course, within the breakdown voltage range, the drive voltage can be increased to further improve the SNR and sensing distance [33].

Table 1 compares the maximum sensing distances of different single pMUTs in the literature. The single PMUT is used as both transmitter and receiver when performing ranging experiments; therefore, it has high requirements for its transmitting and receiving sensitivities. The pMUT designed in this study has a high electromechanical coupling coefficient and amplitude; hence, it can sense a long distance even though it operates at a higher frequency compared to [1,2]. This new pMUT sets a record of 6 m sensing distance, which exceeds the sensing distance of all previously developed single pMUTs.

## 4. Conclusions

In summary, we reported a pMUT that achieves 6 m distance sensing, a record for a long-range sensing pMUTs to date, using a 25% Sc-doped AlN film with a special double-ring structure. The resonant frequency of the pMUT is 91 kHz, and the vibration amplitude at the center of the film reaches 2.87 μm/V, which is about four times higher than that of the conventional structure. The electrical impedance measurements show good electrical performance, with an electromechanical coupling factor of 9.8% for this pMUT. The single pMUT acts as both a transmitter and a receiver, demonstrating its ranging capability. The ranging results demonstrate that the maximum sensing distance of the novel pMUT exceeds that of all previously reported pMUTs. All results considered, this pMUT becomes the most promising alternative to conventional bulk piezoelectric transducers, thus playing an important role in development and functional design, with the aim of achieving more distance sensing applications.

## Figures and Tables

**Table 1:** Comparison of maximum sensing distance.

Reference	Device Type	Piezoelectric Material	Frequency (kHz)	Max Range (m)
[34]	single	AlN	33	1.4
[16]	single	AlN	82	5
This work	single	ScAlN	91	6

## Data Availability

The data presented in this study are available on request from the corresponding author.
